# Effects of Tamoxifen on DNA Integrity in Mice

**Published:** 2019

**Authors:** Sepideh Sadeghi, Ali Reza Talebi, Abbas Shahedi, Mohammad Reza Moein, Abolghasem Abbasi-Sarcheshmeh

**Affiliations:** 1- Department of Biology and Anatomical Sciences, Shahid Sadoughi University of Medical Sciences, Yazd, Iran; 2- Research and Clinical Center for Infertility, Shahid Sadoughi University of Medical Sciences, Yazd, Iran

**Keywords:** DNA, Mice, Sperm, Tamoxifen

## Abstract

**Background:**

Tamoxifen (TX) is widely used to treat idiopathic infertility in men. Using TX has been shown to produce sperm in patients with oligospermia and azoospermia and improve male fertility. The aim of this study was to evaluate the effects of TX on DNA and chromatin quality in mice regarding the importance of chromatin quality and sperm DNA at all stages of reproduction.

**Methods:**

24 male NMRI mice were divided into 3 groups including dose 0.4 *mg/kg/day* that received basal diet and TX, dose 0.6 *mg/kg/day* that received basal diet and TX, and group 3 that received vehicle for 35 days as the control. After that, epididymal spermatozoa were analyzed for nuclear DNA quality*.* One-way ANOVA was performed with a Tukey test to compare sperm DNA fragmentation at different times. The p<0.05 was considered significant.

**Results:**

The use of different doses of TX may have detrimental effects on sperm chromatin protamination and DNA integrity in mice. According to Acridine Orange (AO) staining, the rate of increased single-stranded DNA damage was observed at 0/6 *mg/kg/day* TX dose (p<0.05).

**Conclusion:**

The use of different studied TX doses in the animal sample was found to increase the amount of protamine deficiency and DNA defect in treated mice.

## Introduction

Infertility affects about 8–12% of couples (1) and in 50% of cases, male sex has been involved in infertility (2). TX is a selective non-steroid anti-estrogen receptor molecule which has been usually used to treat male idiopathic infertility (3). Using TX with 20 *mg* dose daily for 6 consecutive months in males with sexual weakness increases the number of ejaculatory sperm (4). In azoospermia, TX can improve sperm count and motility in testicular specimens and it also increases the pregnancy rate in microinjection cycles (5). This kind of drug stimulates the secretion of Follicle-Stimulating Hormone (FSH) and also by blocking the estrogen receptors in the hypothalamus and the pituitary gland, the increased release of the hormone releases gonadotropin.

The important effect of TX on leydig cells and sertoli cells is production of testosterone and support of spermatogenesis, respectively (6). Recent surveys by the American Urology Association have shown that estrogen antagonists are still medically prescribed for clinical treatment of male infertility (7).

Two important families of protamines have been identified in mammals and they have been named protamine 1 (P1), and transition proteins 1, 2 (TP1, TP2) (8). Any disturbances in proteolytic activity to produce protamine 2 (P2) can cause some form of abnormalities in the sperm chromatin structure and cause infertility (9). In normal mode, the ratio of P1 to P2 is approximately equal to one in humans, and any change in this ratio is related to infertility (10). Although the presence of both protamines are essential for the formation of chromatin density, P2 is more effective in this process and its lack is directly related to male infertility (11). A researcher has described the role of chromatin abnormalities and sperm DNA lesions in male infertility in a large study; he has sought to explain factors that cause chromatin abnormalities, such as the lack of proper packaging of chromatin in the nucleus, apoptosis and oxidative stress that if its occurance in more than 30% of sperm leads to fertility decreases significantly. This scholar suggests that chromatin and sperm DNA evaluation should be routinized in In Vitro Fertilization (IVF) labs (12)*.* There is a great deal of association between fragmentation and single-strand DNA with infertility. It has been shown that infertile men suffer from poor health of sperm DNA and the genetic disease is higher in children whose fathers have sperm with a DNA lesion (13).

Today, it has been found that sperm with normal morphology and motility can have chromatin or abnormal DNA. Given the fact that tamoxifen is currently used in male infertility, the effect of tamoxifen was studied on DNA sperm of male mice. Therefore, the aim of present study was to investigate the probable harmful effects of different doses of TX on sperm DNA in mice.

## Methods

In this experimental study, 24 NMRI mice weighting 30–35 *g* with the age of approximately 8 weeks were kept in special cages with easy access to water and food. They used 12 *hr* of light and 12 *hr* of darkness. Mice were divided into 3 groups, two experimental groups were gavaged with TX with doses of 0.4 and 0.6 *mg/kg/day* and one control group was gavaged with distilled water. TX tablets which contained 10 *mg* of this drug were obtained from Iran-Hormone company (Tehran, Iran). It was then suspended in distilled water by sonication and the doses were gavaged to experimental mice daily for 35 days.

After 35 days of treatment for mice, they were sacrificed by cervical spine cord injury, then the right scrotum was dissected and the testicles and the right tail of the epididymis were removed. The sperm were extracted and then kept in the incubator (37°*C*) for 15 *min* by cutting the tail epididymis in each storage of Ham’s F10.

### Sperm DNA evaluation:

Sperm DNA integrity was evaluated using standard cytotoxic techniques including AOT, TUNEL and CMA3 (14). All dyes and chemicals were purchased from Sigma Aldrich Company (St Louis, MO, USA).

### Acridine Orange Test (AOT):

Acridine orange as a fluorescent dye or was used to distinguish the healthy double-stranded DNA from a single-stranded DNA. Acridine orange produces a brilliant green color in response to a double-stranded DNA molecule and regarding a single-stranded DNA molecule, it produces red or orange color (15, 16). After smear and drying in air, fixation was performed in Carnoy’s solution for 2 *hr* at 4°*C*. Coloring was done using the dye of the Acridine orange (0.19 *mg/ml* in McIlvain phosphate-citrate buffer (pH=4)) for 10 *min* in darkness. The evaluation was performed with a 1000× magnification and fluorescent microscope with a 460-*nm* filter (17). At least 200 spermatozoa were counted under fluorescent microscope. Next, the percentage of green (Normal DNA), red (Abnormal DNA) and yellow (DNA with moderate anomaly) sperm in each sample was determined.

### Chromomycin A3 (CMA3) staining:

The fluorescent dye of CMA3 is competing with protamine for binding to minor grooves of DNA which have CG-rich areas and therefore, indirectly shows the amount of protamine deficiency in the chromatin structure. Sperm with protamine deficiency were colored by using 100 *ml* solution of CMA3 and they were observed bright yellow with fluorescence microscopy and were referred to as sperm with immature chromatin (18).

To perform this test, after obtaining smear from each animal sample and drying in air, the slides were fixed in a refrigerator for 10 *min* by fixative Carnoy’s solution and then, using a 100 *μl* solution of CMA3 (Sigma-USA) were stained for 10 *min*. In each slide, at least 200 spermatozoa were counted under fluorescent microscope and an appropriate filter of 470-460 *nm* with a magnification of ×1000 was used (19)*.* The number of sperm (CMA3+) with brilliant yellow color and (CMA3−) sperm without luminosity was determined.

### TUNEL assay:

In this method, the degree of integration of dutp (Deoxyuridine triphosphate) was analyzed into single-stranded and double stranded DNA during TDT-catalysed reaction by fluorescence microscopy or by more accurate flow cytometry. Increasing this integration will increase the amount of damage to DNA. The TUNEL technique is also used to examine the apoptotic process, and the sperm that enter the process are also characterized by having fragmented DNA fragments (20).

After drying the expansions at room temperature, the slides were placed in absolute methanol for 4 *min* and then placed in PBS (×1) for 30 *min*. Subsequently the expansions were incubated in a blocking solution (3% H_2_O_2_ in methanol) for 10 *min* at 15 to 25 °*C,* after washing with PBS for 5 *min*; the expansions were incubated with 0.1% sodium terephthalate 0.1% sodium tetrachloride solution for 2 *min* at 2 to 8 °*C*. The spread was then washed with PBS for 5 *min*. For each slurry, 5 *μl* of enzyme solution and 45 *μl* of labeled solution were mixed into a microtipo and added to all parts of the slide for 1 *hr* in 37°*C* and dark, humid chamber. They were washed with PBS (3 times 5 *min*), mounted with PBS (21). Next, they were observed by fluorescence microscope by magnifying ×1000*.* The number of spermatozoa with bright green nucleus (Apoptotic sperm) and green nucleus without brightness was determined (Normal sperm).

### Statistical analysis:

According to our results, distribution if data was normal and SPSS (Statistical Package for the Social Sciences, version 18.0, SPSS Inc, Chicago, Illinois, USA) software was used to analyze the data. One-way ANOVA was performed with a Tukey test to compare sperm DNA fragmentation at different times. The p<0.05 was considered as the level of significance.

### Ethical consideration:

Animal usage and the protocols were approved by the Institutional Animal Care and Ethical Committee of Biological Sciences of Shahid Sadoughi University of Medical Sciences, Yazd, Iran (IR.SSU.MEDICINE.REC.1395. 99).

## Results

### DNA integrity outcome:

**Figure 1.**
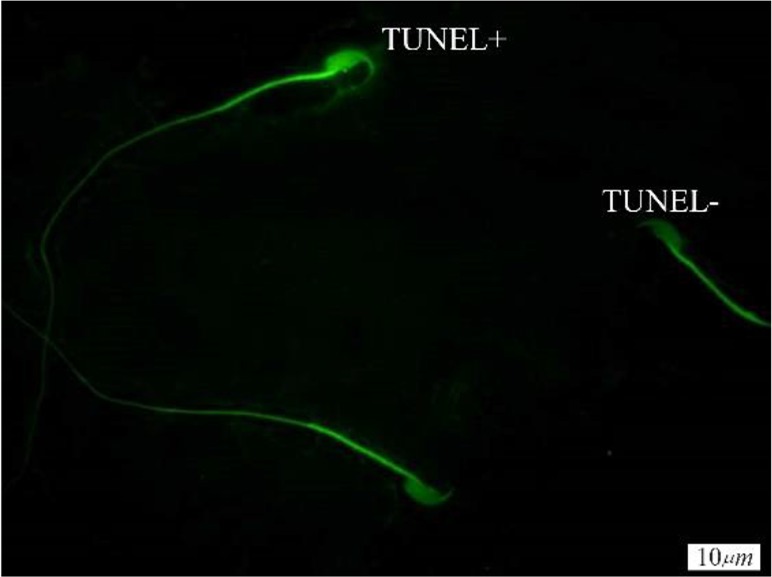
TUNEL staining of spermatozoa; TUNEL+ indicates sperm cells with abnormal DNA and TUNEL− indicates sperm cells with normal DNA (×100 eyepiece magnification)

**Figure 2.**
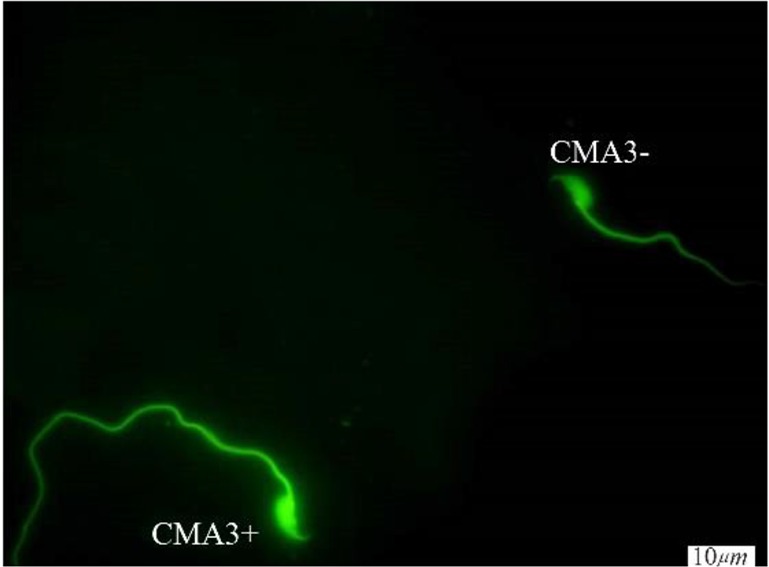
CMA3 staining of spermatozoa; CMA3+ indicates sperm cells with abnormal chromatin and CMA3− indicates sperm cells with normal chromatin (×100 eyepiece magnification)

**Figure 3.**
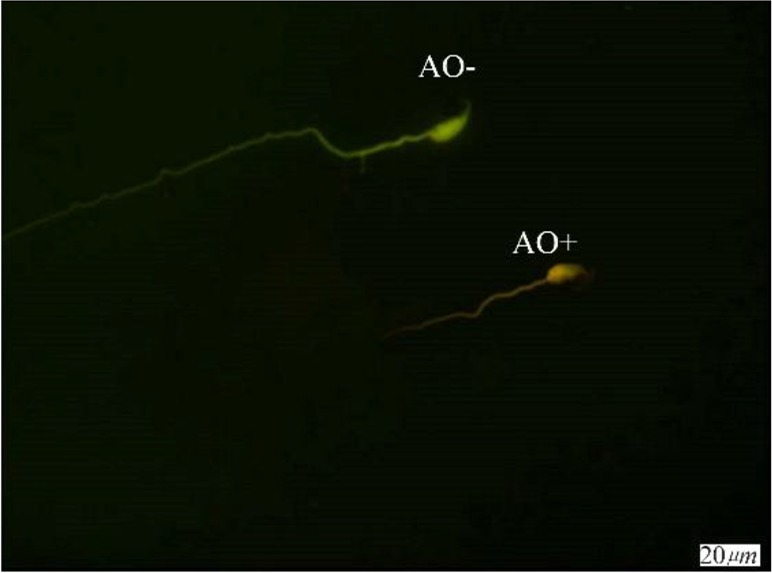
Spermatozoa with native double-stranded DNA (AO−) and denatured DNA (AO+); Acridine Orange staining (×100 eyepiece magnification)

A significant defect increase in DNA integrity was observed in TX treated mice when it was compared with the control group. According to AO, TUNEL and CMA3 staining, there was a significant difference between the three groups. In the study of DNA damage by using the TUNEL, the number of sperm which have been damaged in DNA was increased in I (9.5±3.33) and II (10.37±2.44) groups compared to the III (4.25±1.48) group and there was no difference between groups I and II. TUNEL staining of spermatozoa (TUNEL+) indicates sperm cells with abnormal DNA (Figure 1). According to the CMA3 test, the percentage of sperm with protamine defect increased in group I (14.62±4.53) and II (10.37±2.44) in comparison to group III (9.0±2.07) and there was no difference between groups I and II (Figure 2). Significant differences were observed in the increase of denaturation of DNA by AO staining in group II (10.37±2.4) in comparison to group I (7.75±2.6) and III (7.75±1.7) and there was no difference between groups I and III (Table 1). The figure 3 shows one sperm with native double-stranded DNA (AO−) and one sperm with denatured single-stranded DNA (AO+).

**Table 1.** The results of sperm analysis in two experimental groups; dose 0.6 *mg/kg/day* (I) and dose 0/4 *mg/kg/day* (II) and control group (III)

**Group**	**Group I (0/4 *mg/kg/day*)**	**Group II (0/6 *mg/kg/day*)**	**Group III (control)**	**p-value group I**	**p-value group II**	**p-value group III**	**p-value total**
**AO**	7.750±2.604	10.375±2.445	7.750±1.752	1.00	0.08^#^	0.08	0.045
**CMA3**	14.625±4.533	10.375±2.445	9.00±2.070	0.01^*^	0.001^*^	0.1	0.001
**TUNEL**	9.50±3.338	10.375±2.445	4.250±1.488	0.001^*^	0.005 ^*^	0.8	0.001

The results are shown as mean±SD (p<0.05. For the analysis of data, one way ANOVA was used. AO: Acridine orange, CMA3: Chromomycin A3, TUNEL.

* Significant difference between group III and the two experimental groups on CMA3 and TUNEL staining.

# Significant difference between group II with groups I and III on AO staining

## Discussion

The main finding of the present study shows that taking different doses of tamoxifen weakens sperm DNA quality and sperm chromatin. In Assisted Reproduction Technology (ART), there is a direct relationship between sperm DNA integrity and fertility results. It is shown that in cases of sperm DNA anomalies above 12%, none of the oocyte will be fertilized by abnormal spermatozoa (22) and as a result, IVF is failed. Intracytoplasmic Sperm Injection (ICSI) and Intra Uterine Insemination (IUI) cycles are done in both human and animals (23). Increasing the rate of sperm single-stranded DNA in men with lower fertility can be related to the defects in the epididymal and testicular function (24). The data from the present study showed that the use of different doses of TX may increase the rates of sperm chromatin/DNA anomalies in mice spermatozoa. In a study, the beneficial effects of TX on sperm maturation have been shown in infertile men with non-obstructive azoospermia and it was concluded that TX can increase the number of spermatozoa in those patients (5). On the other hand, another study has shown that the use of TX increases the thickness of testicular tunica albuginea and decreases the diameter and thickness of the seminiferous tubules. They also showed that TX decreases the number of spermatogonia type A, spermatogonia type B, primary spermatocyte, spermatid, sperm, sertoli and leydig cells (25)*.* One study showed that although the daily use of TX reduces the epididymis sperm motility, it cannot affect other sperm parameters in rats. They also showed that TX causes a significant reduction in fertility potential and it can increase the rate of embryonic damage before and after implantation. In our study, tamoxifen had negative effects on the quality of sperm DNA, which may have negative effects on fertility (26). A more recent study was done by Nada et al. about the effects of TX on sperm structure and levels of oxidative stress in idiopathic oligoasthenoteratospermic (OAT) patients. They have confirmed that the consumption of TX can significantly improve the level of mal-ondialdehye (MDA), sperm concentration, sperm morphology, acrosome and mitochondrial malformations (27). However, it is clear that consumption of the estrogen antagonists drugs such as TX, can improve the chance of fertility specially count of spermatozoa in infertile patients with low serious side effects (28).

On the other hand, our results determined some anomalies in sperm chromatin and DNA following TX administration. To explain the mechanisms of TX action on sperm cells, it is shown that the use of this drug significantly reduces the DNA methylation of the specific DNA methylation in rat spermatozoa (IGF2-H19) region in spermatozoa (29). Experiments showed that natural methylation in normal spermatogenesis and embryos is very important in men's germ production line (30). Insulin-like growth factor 2 (Igf2) mediates mitogenic reactions via Igf1, and it can carry out fetal development on days 9-11 of pregnancy in rodent (31). Recent studies have shown that hypomethylation of Igf2-H19 in spermatozoa of men with oligozoospermia impair the spermatogenesis, which may lead to abnormal growth of the fetus (32, 33).

Another molecular mechanism of TX action on sperm chromatin was shown by Aleem et al. They have concluded that the Tamoxifen citrate reduces the level of sperm chromatin condensation by decreasing testicular levels of TP1, TP2, protamine 1 and response element modulator-τ (CREMs) proteins, which are effective in chromatin compaction in the spermatogenesis process, and also TX can affect the expression of these genes in both levels of transcription and post-translational modifications (34)*.*

## Conclusion

Although, the TX increase the count of spermatozoa, our results showed that the different doses of TX may have harmful effects on sperm chromatin quality and DNA integrity in mouse spermatozoa as an experimental model. further studies are needed to evaluate the molecular effects of TX on other sperm structures.
